# Alkyl Chain Grafted-Reduced Graphene Oxide Membrane for Effective Separation of Water/Alcohol Miscible Mixtures

**DOI:** 10.3389/fchem.2020.598562

**Published:** 2020-12-03

**Authors:** Hailong Zhang, Jianbo Yang, Ting Li, Xingxiang Ji, Zhen Xu, Yaling Zhu, Libin Liu

**Affiliations:** State Key Laboratory of Biobased Material and Green Papermaking, School of Chemistry and Chemical Engineering, Qilu University of Technology (Shandong Academy of Sciences), Jinan, China

**Keywords:** reduced graphene oxide, membrane, alkyl chain, water/alcohol separation, filitration

## Abstract

Separation of water/alcohol miscible mixtures via direct filtration only under gravity is a great challenge. Here, different alkyl chain grafted-reduced graphene oxide (alkyl-RGO) is synthesized and characterized. The hydrophobic alkyl chains can considerably modify the oil-wettability of the membranes and avoid water permeation. The alkyl-RGO membrane obtained by vacuum filtration can separate water/oil immiscible mixtures. Importantly, water/alcohol miscible mixtures could also be separated solely under gravity, where alcohols efficiently permeate the alkyl-RGO membrane while water is prevented through the membrane. The separation efficiency of C_12_H-RGO membrane reaches up to about 0.04 vol% of water content for the case of separating an n-propanol/water (90:10 v/v) mixture with high n-propanol permeability of approx. 685 mL m^−2^ h^−1^. Molecular simulations indicate that the selective absorption ability and diffusion rate also affect water/alcohol separation. The alkyl-RGO membranes via gravity driven filtration can extend the applications of separation of water/alcohol miscible mixtures.

## Introduction

Separation and purification of alcohol from water/alcohol mixtures are very important due to their wide application in the chemical industry or as oil-based fuels (Nguyen et al., 2020; Sapegin et al., 2020). Generally, alcohols and water mixtures often form azeotropes or are similar in their physicochemical properties, making their separation particularly difficult. To solve this problem, several strategies including liquid–liquid extraction, pervaporation, and adsorption have been used as potential separation techniques (Zhao et al., 2020). Among them, membrane-based pervaporation technology has been recognized as a good substitute for conventional energy-intensive separation processes and has been studied intensively (Zuo and Chung, 2013; Lively and Sholl, 2017; Ying et al., 2017; Zhang et al., 2020). For example, Jiang et al. reported the enhanced separation selectivity by an efficient mussel-inspired approach during the pervaporation process (Zhao et al., 2015). However, complicated processes are required in the pervaporation, such as vacuum equipment and heating and cooling devices. Previously, membrane-based separation for oil/water mixtures via direct filtration has been extensively reported by Jiang (Qu et al., 2019), Seeger (Chu et al., 2015), Tuteja (Tuteja et al., 2007; Kota et al., 2012), and others (Cao et al., 2019; Wang et al., 2019; Yang et al., 2019). Our group has also demonstrated a pH responsive coating and self-healing electrospun membrane for the separation of oil and water mixtures (Dang et al., 2016; Fang et al., 2016). All of these achievements are based on designing materials with special wettability that are superhydrophobic (Wang B. et al., 2015; Wang H. et al., 2015; Ge et al., 2019) or under-water superolephobic (Dudchenko et al., 2015; Gao et al., 2016). However, alcohol and water are miscible and all of these membranes are not suitable for water/alcohol mixture separation. Therefore, direct separation of water/alcohol mixtures by membrane-based filtration is a great challenge.

Due to their unique two-dimensional (2D) structures and adjustable nanopores, graphene or its derivatives including graphene oxide (GO) or reduced graphene oxide nanosheets, have great applications in the field of gas or liquid separation (Kim et al., 2013; Li et al., 2013; Niu et al., 2014; Liu H. et al., 2015; Sun et al., 2016; Kidambi et al., 2017; Wei et al., 2017; Xu et al., 2017; Ling et al., 2020). For example, Geim et al. (Nair et al., 2012) reported that micrometer-thick GO membranes allowed unimpeded percolation of water, but impeded liquids, vapors and gases. Li and co-workers (Qiu et al., 2011) constructed a type of wet graphene membranes for the separation of nanoparticles and dyes. Gao et al. (Han et al., 2013) reported that base-refluxing reduced GO membranes could separate organic dyes and water mixtures. In addition, the diverse interplays between ions and GO or reduced GO gave rise to different interaction strengths, which resulted in excellent selectivity of the membranes toward various ion species in solutions when permeating through membranes (Cohentanugi and Grossman, 2012). Besides water purification and ion selectivity, RGO-based membranes for organic solvent filtration have also been reported (Huang et al., 2015, 2016). In brief, all of these achievements illustrated that the 2D nanochannels between GO or reduced GO membranes can provide pathways for gas, ion and organic solvent separation as well as water desalination based on size-dependent molecular sieving and diverse interactions.

Separation of miscible water/organic solutions by graphene-related materials has also been reported, which is still based on the pervaporation process. For example, a ceramic hollow fiber coated by GO membrane exhibited excellent water permeation for dimethyl carbonate/water mixtures through a pervaporation process (Huang et al., 2014). Gorgojo et al. (Alberto et al., 2016) reported organophilic mixed matrix membranes containing graphene-like fillers for separation of 1-butanol and ethanol from aqueous solutions. Recently, Pan et al. (Zhang et al., 2018) reported that polydopamine grafted GO composite membranes could separate 70 wt% ethanol/H_2_O mixture and 70 wt% isopropyl alcohol/H_2_O mixture by pervaporation, respectively. More recently, Wang et al. (Chen et al., 2020) reported robust angstrom-channel graphene membranes which can concentrate ethanol to 99.9 wt% from dilute solution with one to two orders of flux than conventional pervaporation membranes. However, separation of miscible water/alcohol mixtures via a graphene-based membrane filtration technology only by gravity has been seldom reported.

**Figure 1:**
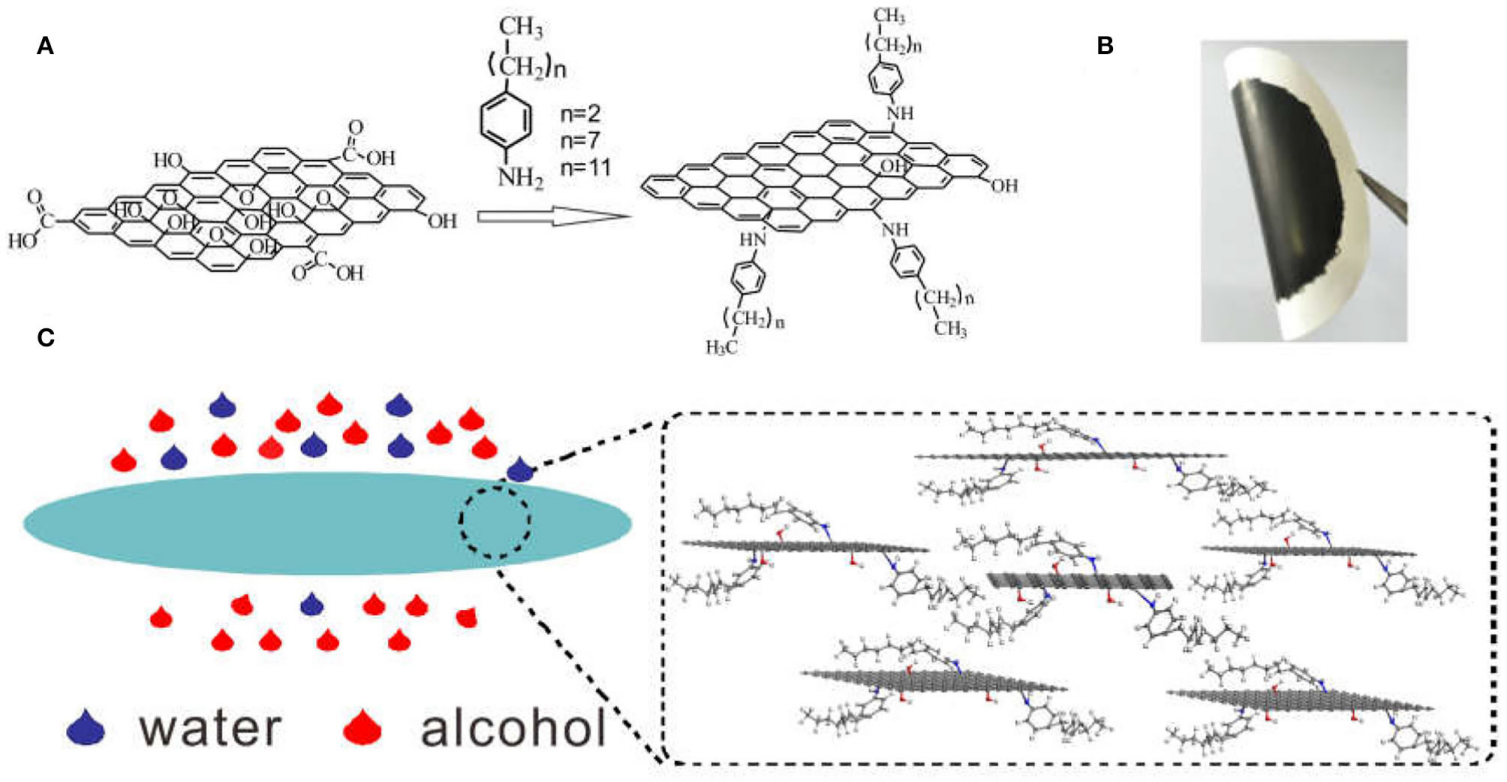
**(A)** Schematic of the reaction of GO with different alkylanilines. **(B)** Photograph of the alkyl-RGO membrane on a cellulose ester membrane via vacuum filtration. **(C)** Schematic illustration of the alkyl-RGO membrane for passing through alcohol while blocking water.

Herein, we provide an alternative strategy by using a facial filtration process to separate water/alcohol via a RGO-based membrane. Considering that the hydrophilic GO can be reduced to hydrophobic RGO by reduction, which may hinder water permeation, different alkyl chain (n-propyl, n-octyl and n-dodecyl) grafted RGO (here referred to as C_3_H-RGO, C_8_H-RGO and C_12_H-RGO, respectively) were designed and fabricated through simultaneous reduction and grafting on GO by the corresponding alkylaniline (Figure 1A). Thus, the alkyl chain may enlarge the interplanar distance of the reduced GO, and the corresponding alkyl-RGO membranes fabricated by vacuum filtration can permeate alcohol while blocking water permeation solely by gravity (Figures 1B,C). The molecular simulation indicates that the absorption and diffusion of water and alcohol are different when passing through the membrane.

## Materials and Methods

### Materials

Natural graphite flakes (8,000 mesh, 99.95% purity), p-propylaniline, p-n-octylaniline, and p-n-dodecylaniline were supplied by Aladdin. Concentrated sulfuric acid (95–98%), concentrated hydrochloric acid (36–38%), potassium permanganate, and solvents used in this work were all analytically pure and purchased from Beijing Chemical Factory (China). Hydrogen peroxide (H_2_O_2_) and sodium nitrate were supplied by LaiYang Shi Kant chemical company. Cellulose ester membrane was supplied by Hangzhou Special Paper Co. Ltd.

### Preparation of Alkylaniline Functionalized RGO (alkyl-RGO)

Graphene oxide (GO) was obtained from natural graphite by the Hummers method (see Supplementary Material). GO (0.6 g) and p-propylaniline (0.9 g) was mixed in 90 mL of ethanol in a three-neck flask. The mixture was refluxed at 100°C for 12 h with stirring. Then, the resulting solution was filtrated with a polypropylene membrane (~0.22 μm). The collected powders were rinsed by ethanol and filtrated to remove the physically absorbed p-propylaniline. Finally, the product (referred to as C_3_H-RGO) was dried in an oven at 80°C for 24 h. We also obtained p-n-octylaniline and p-n-dodecylaniline functionalized RGO (referred to as C_8_H-RGO and C_12_H-RGO, respectively) with the same procedure.

### Fabrication of Alkyl-RGO Membranes With Different Thicknesses

Alkyl-RGO membranes were fabricated via vacuum filtration of dispersions of C_3_H-RGO, C_8_H-RGO, and C_12_H-RGO in dimethylformamide (DMF) (0.1 mg/mL, 0.15 mg/mL, 0.2 mg/mL), respectively. Each membrane was fabricated by filtrating different volumes of dispersions on a cellulose ester membrane (50 mm diameter, 0.22 μm pore size). During the filtration process, the vacuum degree is kept at nearly−0.1 MPa. Finally, the membranes were dried in an oven at 50°C for more than 12 h until the mass of the membranes did not change (TGA was used to confirm no DMF left, not shown). Thus, alkyl-RGO membranes with different thicknesses were obtained.

### Oil/Water Separation Experiment

#### Separation of Immiscible Mixtures

Several organic solvents including diesel, petroleum ether, toluene, n-hexane, bromoethane, and dichloromethane were used to mix with water at a volume ratio of 1:1 for separation. When the mixed solutions were poured into the separation device, the oil passed through while water was retained on the membranes, thus achieving water and oil separation. The flux was measured by pouring 30 mL of various types of oil/water mixtures into the separation device. The time of the permeate passing through the device was recorded and the separation flux was calculated as the following equation:

(1) $$\begin{array}{r} \text{flux}=\frac{V}{St} \end{array}$$

where V is the volume of the permeate, *S* is the valid area of the membrane and *t* is the testing time. After each separation, the membrane was simply washed by ethanol and dried. The recovery was determined by calculating the ratio of the volume of collected organic liquids to the volume of the organic feed.

#### Separation of Miscible Water/Alcohol Mixtures

Using the same separation device for the separation of water miscible liquids including methanol, ethanol, n-propanol, isopropanol and DMF, the mixed solutions (30 mL) with 10% volume fraction of water were poured into the separation device without pressure and only under gravity. The effective membrane area is π(0.02 m)^2^. At different times, the compositions of the permeate were determined by the gas chromatographic technique using a flame ionization detector. The separation factor α was determined by the following equation:

(2) $$\begin{array}{r} \alpha =\left(Yalcohol/Ywater\right)/\left(Xalcohol/Xwater\right) \end{array}$$

Here, *X* and *Y* are the volume fractions of alcohol and water in the feed and permeate sides, respectively.

### Gas Chromatography Measurement

The permeate composition after the separation of water miscible solution was detected by a GC-9860, SE-30 column with a length of 30 m. The temperatures of the gasify room and column were 200 and 160°C, respectively. By using cyclohexanone as the internal standard substance, the water content could be obtained by calculating the ratio of the integral areas of the ethanol and internal standard. The detailed calculation method is described in the Supplementary Material.

### Molecular Dynamic Simulation

The molecular dynamic simulation of all systems can be performed after charges and potentials are assigned to each atom. The following assumptions were made during the simulation: (1) The simple point charge (SPC) model is adopted for water molecules of the solutions. (2) The long-range electrostatic interactions are accounted for using the Ewald method. (3) Periodic boundary conditions are adopted in three dimensional directions of the system. (4) The simulation process is isothermal.

The total energy is written as a combination of valence terms including diagonal and off-diagonal cross-coupling terms and non-bond interaction terms, which include the Coulombic and Lennard-Jones functions for electrostatic and van der Waals interactions,

(3) $$\begin{array}{r} E=E_{\text{bonds}}+E_{\text{angles}}+E_{\text{dihedrals}}+E_{\text{cross}}+E_{\text{VDW}}+E_{\text{elec}} \end{array}$$

where *E*_VDW_ and *E*_elec_ are given by Equation (4):

(4) $$\begin{array}{r} E_{\text{non}-\text{bond}}=E_{\text{VDW}}+E_{\text{elec}}=\sum \varepsilon_{ij}\left[2{\left(\frac{\sigma_{ij}}{r_{ij}}\right)}^{9}-3{\left(\frac{\sigma_{ij}}{r_{ij}}\right)}^{6}\right]+\sum \frac{q_{i}q_{j}}{r_{ij}} \end{array}$$

The parameters for each like-site interaction are given by the COMPASS force field (Sun, 1998; Sun et al., 1998). All three alkyl-RGO structures are constrained during the simulation. The energies of the initial configurations are minimized with the Smart Minimizer method. After the minimization, all simulations are equilibrated at constant temperature (273 K) and volume (NVT) for 5 ns. Atomic coordinates are saved every 20 ps. The analysis is performed by averaging over the final 1 ns of each trajectory.

The absorbed energy of ethanol is calculated as follows:

(5) $$\begin{array}{r} E_{\text{alcohol}}=\left[E_{\text{total}}-\left(E_{\text{solution}}+E_{\text{SAMs}}\right)\right]/N_{\text{alcohol}} \end{array}$$

where *N*_alcohol_ is the number of alcohol molecules, *E*_solution_ is the interaction energy of the solution, *E*_total_ is the potential energy of the energy-minimized system in equilibrium, and *E*_SAMs_ is the potential energy of the single alkyl-RGO monolayers.

The mean square displacements (MSD) of water and alcohol molecules in three systems are calculated from Equation (6):

(6) $$\begin{array}{r} MSD\left(t\right)=\langle \frac{1}{N}\overset{N}{\underset{i=1}{\sum }}{\left|r_{i}\left(t\right)-r_{i}\left(0\right)\right|}^{2}\rangle \end{array}$$

where *N* is the number of target molecules and *r*_i_(*t*) is the position of molecule *i* at time *t*. This figure displays the MSD of the water and alcohol molecules in the final 1,000 ps of the final equilibrium trajectory. Diffusion coefficients (D) can then be obtained from the slope of the mean square displacement vs. time curve, using the well-known Einstein relation,

(7) $$\begin{array}{r} D_{\alpha }=\frac{1}{6N_{\alpha }}\underset{t\to \infty }{\lim}\frac{d}{dt}\overset{N_{\alpha }}{\underset{i=1}{\sum }}\langle {\left[r_{i}\left(t\right)-r_{i}\left(0\right)\right]}^{2}\rangle \end{array}$$

where *d* is the dimensionality of the system, and *r*_i_(*t*) and *r*_i_(*0*) are the center-of-mass coordinates of the *i*th molecules at times *t* and *t* = 0, respectively.

### Characterization

FTIR spectra were obtained on an IR Prestige-21 FTIR spectrometer (Shimadzu, Japan). X-ray diffraction (XRD) analyses were carried out on a D-8 ADVANCE X-ray diffractometer (Bruker AXS, Germany). Alkyl-RGO and GO powders dried from aqueous solution were used to measure XRD. X-ray photoelectron spectroscopy (XPS) measurements were conducted in an ESCALAB 250 (Thermo Fisher Scientific, America) using a monochromatic Al-Kα X-ray source at 100 W. Scanning electron microscope (SEM) images were obtained by a QUANTA 200 (FEI, America). The thickness of the alkyl-RGO membranes was obtained by scanning at least three different samples and the average values were used to evaluate the thickness. The Raman spectra were obtained using a LabRAM HR800 Raman spectrometer (HORIBA JY, France). The equilibrium contact angles (CA) were measured by a DSA 100 (KRÜSS, Germany) contact angle meter at ambient temperature. Atomic force microscopy (AFM) images were carried out on a Mutimode 8 Nanoscope V system (Bruker, USA) in peak force tapping mode. Different alkyl-RGO membranes were measured by AFM and the surface roughness were obtained by scanning at least three different areas.

## Results and Discussion

### Characterization of Alkyl-RGOs

**Figure 2:**
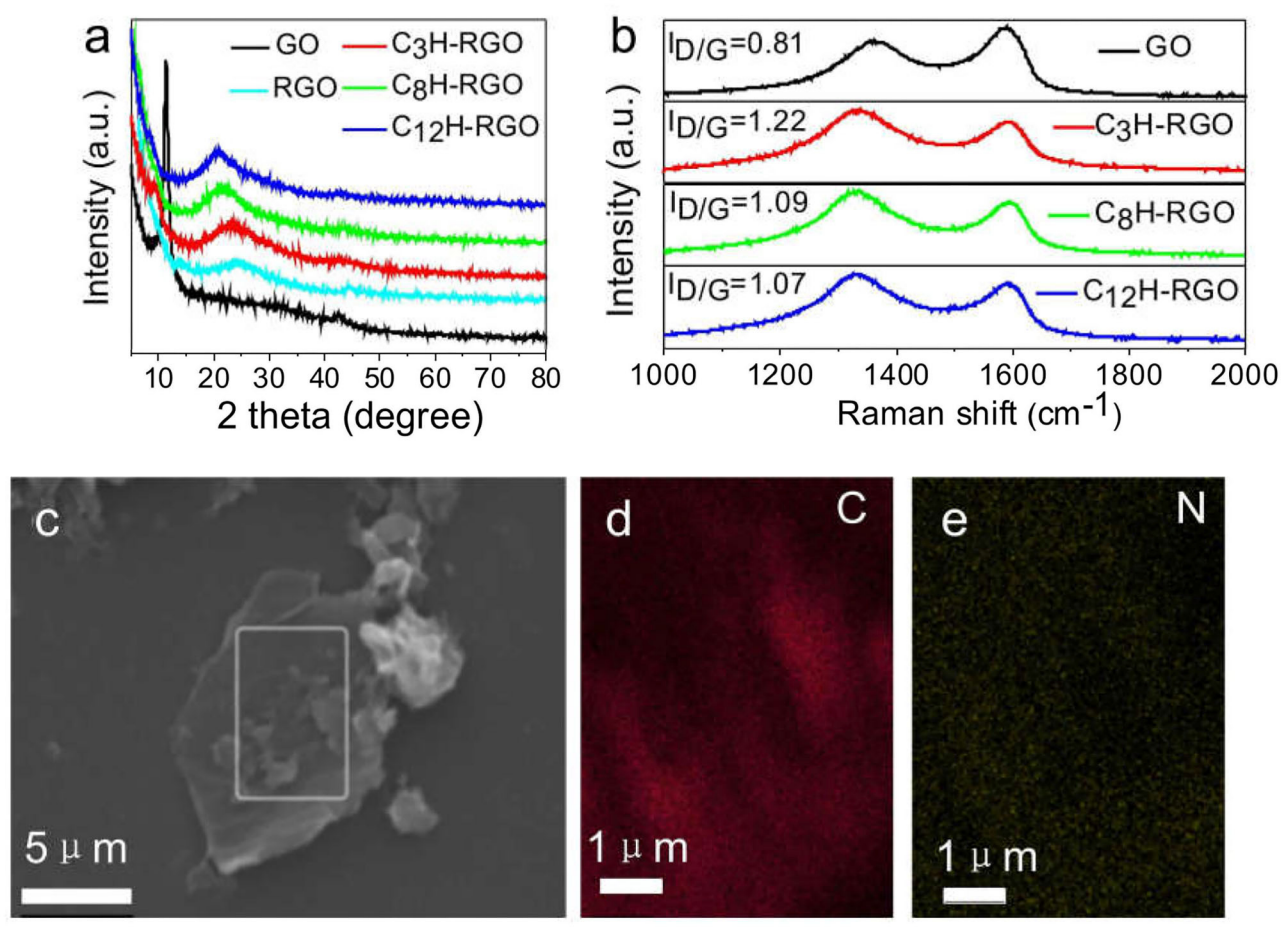
Characterization of alkyl-RGO. **(a)** XRD spectra of alkyl-RGO, GO, and RGO (obtained by hydrazine reduction). **(b)** Raman patterns of GO and different alkyl chain grafted-RGO. **(c)** SEM image of C_12_H-RGO and **(d,e)** elemental mapping of C_12_H-RGO in **(c)** for C and N, respectively.

The different alkyl-RGOs were synthesized according to a previous report (Li et al., 2015) (Figure 1A). After filtration, the alkyl-RGO layer could tightly adhere to the cellulose ester membrane. We tried to uncover the layer from the cellulose ester membrane, but failed. The composite membranes are flexible as shown in Figure 1B. In the FTIR spectra, two new peaks centered at 2,921 cm^−1^ and 2,842 cm^−1^ appear for alkyl chain grafted-RGO compared to that of GO, which are assigned to the stretching vibration of CH_2_. In addition, the NH stretching peak at 1,564 cm^−1^ indicates the formation of the C-NH-C bond (Supplementary Figure 1). All of these changes indicate the successful grafting of alkyl chains on the GO sheets. The reduction of GO by alkylaniline is proved by XRD measurement (Figure 2a). The alkyl-RGOs exhibit a weak and broad diffraction peak centered at 23.32°, 21.32°, and 21.15° with an interplanar distance of 3.81, 4.16, and 4.20 Å for C_3_H-RGO,C_8_H-RGO, and C_12_H-RGO, respectively. These interplanar distances of alkyl-RGO are slightly higher than that of graphite (3.35 Å) and much lower than that of GO precursor (7.78 Å), indicating the successful reduction of GO into RGO sheets. Considering a large amount of oxygen-containing group on GO, we fabricated RGO by hydrazine reduction of GO. As expected, the interplanar distance of alkyl-RGO is slightly larger than that of RGO obtained by hydrazine reduction, indicating the successful reduction and grafting of alkyl chains on GO sheets. In addition, the interplanar distance of alkyl-RGO also increases as the alkyl chain length increases, which may affect the alcohol permeation. The Raman spectra indicate that two bands around 1,349 cm^−1^ and 1,583 cm^−1^ are assigned to the D-band and G-band of carbon, respectively (Figure 2b). The intensity ratio of D-band and G-band (I_D_/I_G_) are calculated to be 1.22, 1.09, and 1.07 for C_3_H-RGO, C_8_H-RGO, and C_12_H-RGO, respectively, which are higher than that of GO (0.81), confirming that the GO sheets are reduced and that some conjugated structure of GO is converted to single bonds (Li et al., 2015; Liu J. et al., 2015). The surface chemistry of C_12_H-RGO was further characterized by X-ray photoelectron spectroscopy (Supplementary Figure 2). A new element (N) appears in the C_12_H-RGO spectra. The molar ratio of C/O is about 4.2 for C_12_H-RGO, which is much higher than that of GO (2.1) and slightly lower than that of RGO obtained by hydrazine reduction (5.2) (Supplementary Figure 3). The C 1s spectra of GO and C_12_H-RGO also reflected the reduction process. For GO, four different peaks centered at 284.6, 286.6, 287.2, and 288.5 eV correspond to C–C in the unoxidized graphite carbon skeleton, C–OH in the hydroxyl group, C–O–C in the epoxide group, and O–C=O in the carboxyl group, respectively (Li et al., 2015; Yang et al., 2020) (Supplementary Figure 2b). After reduction by dodecylaniline, the peaks corresponding to the oxygen-containing groups of C_12_H-RGO are significantly weakened, indicating that a large number of oxygen-containing groups have been removed (Supplementary Figure 2c). The elemental mapping confirms that the alkyl chain is uniformly distributed on the RGO sheet (Figures 2c–e).

**Figure 3:**
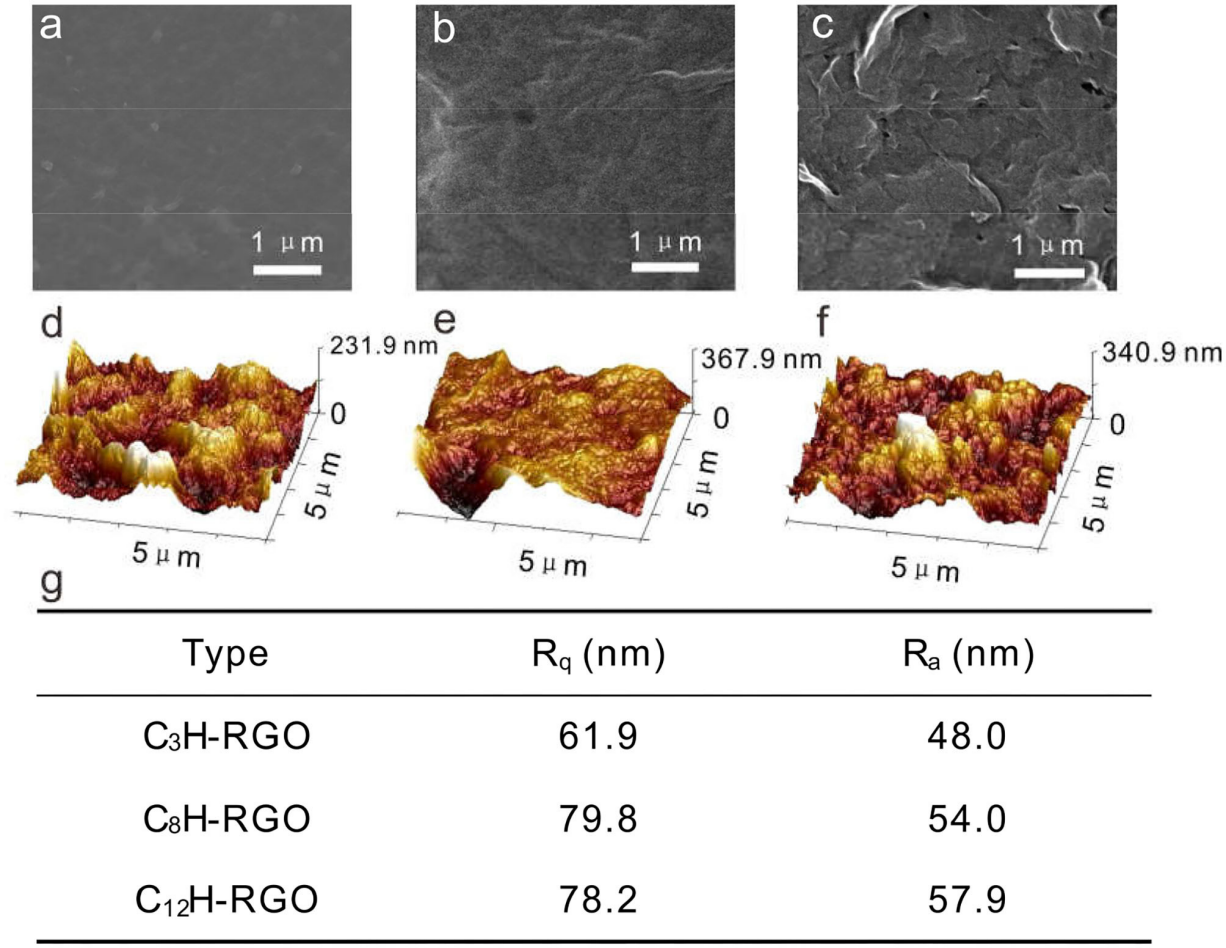
SEM images **(a–c)** and AFM images **(d–f)** of C_3_H-RGO **(a,d)**, C_8_H-RGO **(b,e)** and C_12_H-RGO **(c,f)** with thicknesses of 0.93, 0.58, and 0.49 μm, respectively. **(g)** Root mean square roughness (R_q_) and average roughness (R_a_) of the corresponding membranes from AFM height images.

**Figure 4:**
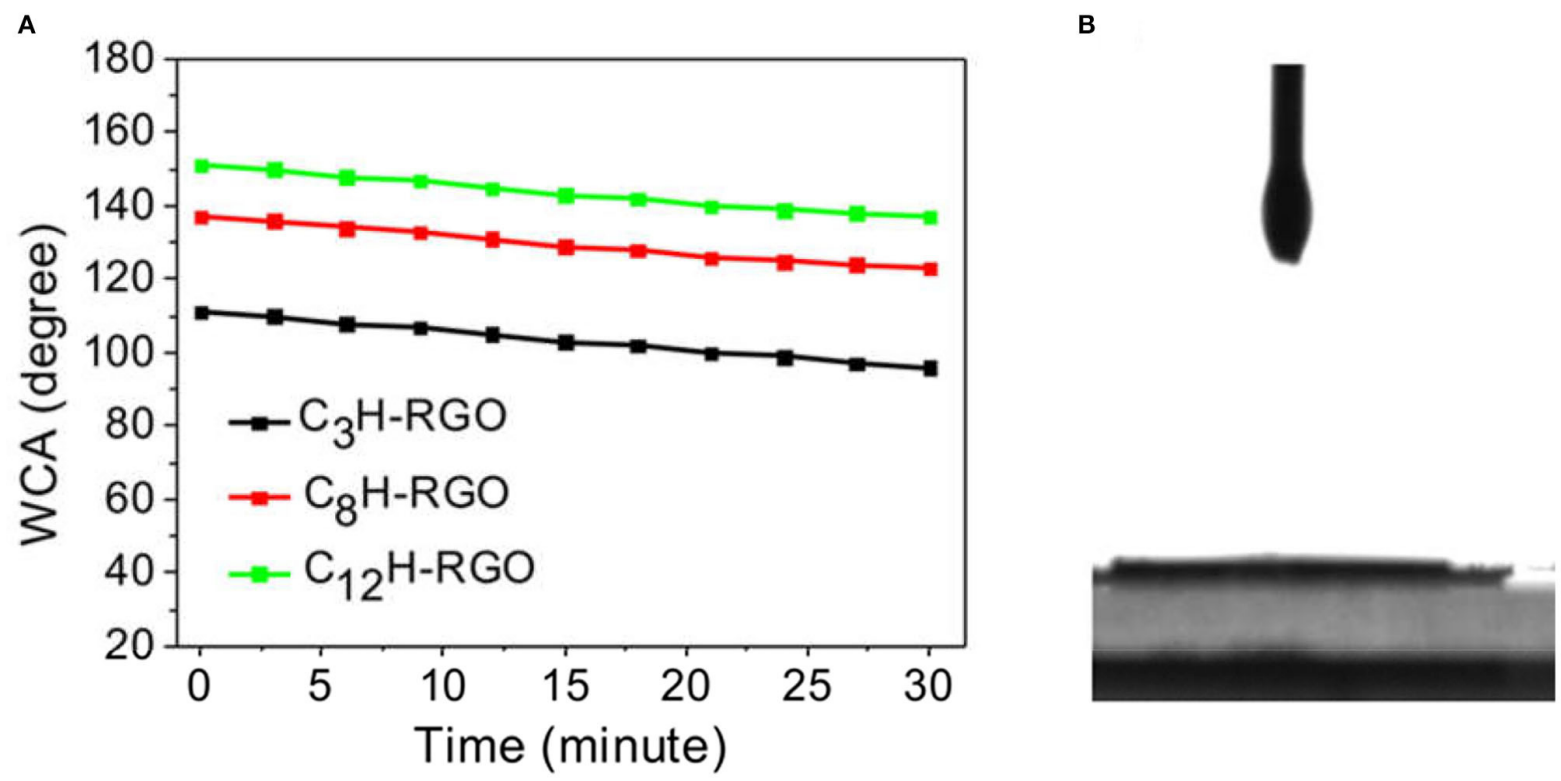
**(A)** Changes in the water contact angle (WCA) with time for different alkyl chain-modified RGO membranes. **(B)** Hexane droplets on C_12_H-RGO membranes.

The alkyl-RGO membranes were first tested for water/oil separation. The key point of oil/water separation is the special surface wettability of membranes. Therefore, the surface wettability and morphology of the membrane were first characterized. The alkyl-RGO membranes were obtained by facial vacuum filtration of alkyl-RGO in DMF dispersions. Three alkyl-RGO membranes (C_3_H-RGO ~ 0.93 μm, C_8_H-RGO ~ 0.58 μm, and C_12_H-RGO ~ 0.49 μm) were measured by SEM and AFM, respectively. As characterized by SEM (Figures 3a–c), the surfaces of C_3_H-RGO and C_8_H-RGO are smoother and no obvious corrugations are observed, while the surface of C_12_H-RGO reveals significant folding of RGO sheets. The root mean square roughness and the average roughness obtained from AFM height images are all obviously increased as length of the alkyl chains increases from C3 to C8 and C12, respectively (Figures 3d–g). This is consistent with the water contact angle measurement, which shows that the contact angle is increased from 100 ± 3° for C_3_H-RGO membrane to 150 ± 3° for C_12_H-RGO membrane. In addition, the stability of the water droplet on the alkyl-RGO membrane is slightly decreased. After 30 min, the water contact angles still remain in the hydrophobic range of 137 ± 3° for the C_12_H-RGO membranes (Figure 4A). When oil (e.g., n-hexane) is dropped on the membrane surface, it immediately penetrates into the films, which indicates the high oleophilicity (Figure 4B). It is noted that the longer alkyl chains on the RGO sheet endow the membranes with more roughness and a larger contact angle, which facilitate the water/immiscible liquid separation and water/alcohol separation (see below).

### Separation of Immiscible Oil/Water Mixtures

**Figure 5:**
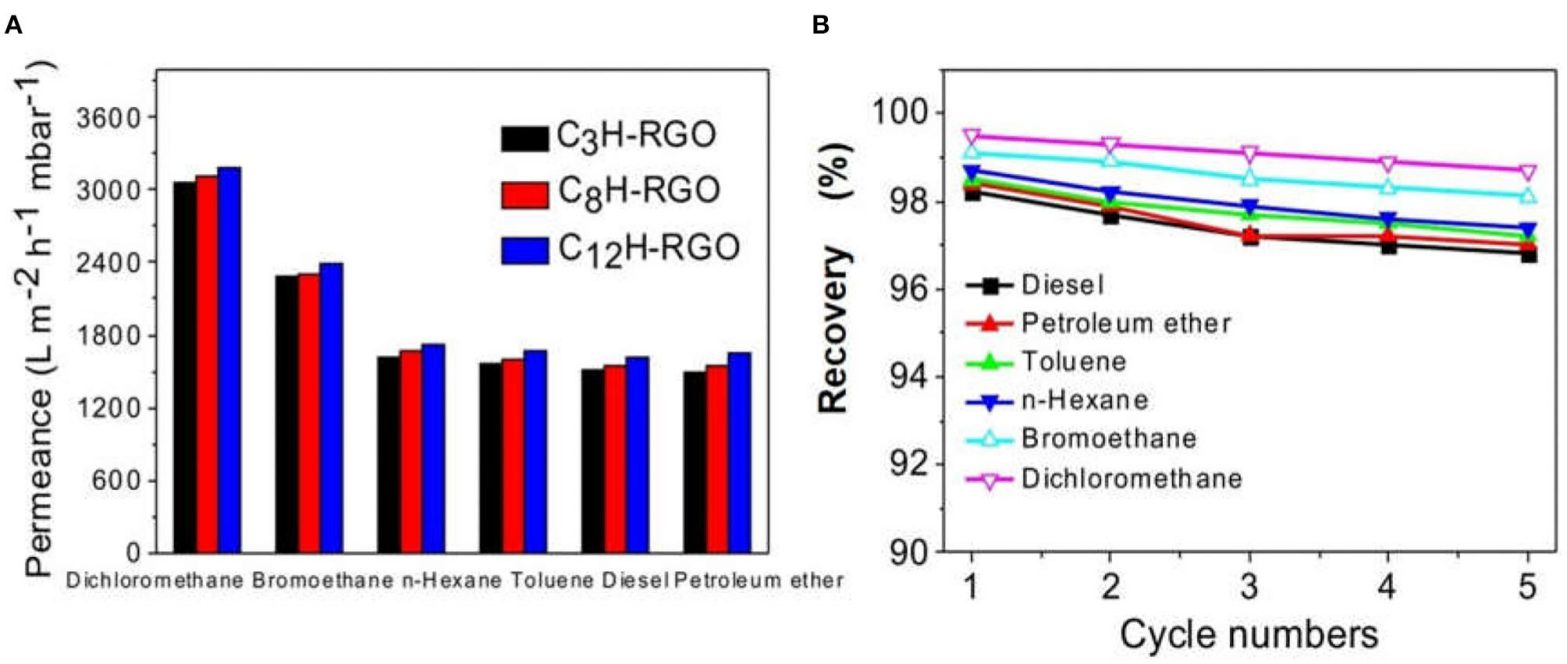
**(A)** Permeate flux of different oil/water mixtures for C_3_H-RGO, C_8_H-RGO, and C_12_H-RGO membranes. **(B)** Recovery for C_12_H-RGO membranes after 5 cycles.

For some traditional membranes, the surface wettability for water and oil is not distinctly different. When absorbing oil, the membrane takes in water simultaneously and vice versa, which results in a decrease in the separation efficiency (Kota et al., 2012). In our case, the superhydrophobic surface is favorable for water/oil separation. The thickness of the membrane can be easily controlled by varying the volume and concentration of the dispersion. Thus, alkyl chain grafted-RGO membranes with different thicknesses have been fabricated (Supplementary Figure 4). We selected C_3_H-RGO, C_8_H-RGO, and C_12_H-RGO membrane with thicknesses of 0.93 μm, 0.58 μm, and 0.49 μm, respectively, to measure the oil/water separation. Several organic solvents including diesel, petroleum ether, toluene, n-hexane, bromoethane, and dichloromethane were used to mix with water at a volume ratio of 1:1 for separation. For all of the alkyl-RGO membranes, quite high fluxes are obtained as shown in Figure 5A. For example, the obtained fluxes of C_12_H-RGO membranes were 3,184 ± 36, 2,388 ± 30, 1,719 ± 26, 1,671 ± 25, 1,624 ± 20, and 1,643 ± 28 Lm^−2^h^−1^mbar^−1^ for dichloromethane, bromoethane, n-hexane, toluene, petroleum ether, and diesel, respectively. The recovery was determined by calculating the ratio of the volume of collected organic liquids to the volume of the organic feed. For all alkyl chain-grafted RGO membranes, the recovery is higher than 98%. After 5 recycles, the recovery is still higher than 97% (Figure 5B).

In our case, the recovery may be related to the density of the separated oil. The densities of dichloromethane and bromoethane are 1.33 and 1.47 g cm^−3^, respectively; while the densities of the other studied oil are lower than 1 (Supplementary Table 1). Thus, the obvious higher recovery for dichloromethane and bromoethane should be due to their higher densities (Xi et al., 2019).

### Separation of Water Miscible Mixtures

More importantly, the alkyl chain-grafted RGO membranes can also separate water/alcohol miscible solution. After miscible solutions containing 10 vol% water and 90 vol% different liquids such as methanol, ethanol, n-propanol, isopropanol, and DMF were filtrated through the membranes, the compositions of the permeate were determined by gas chromatography (see Experiment section and Supplementary Material). It is found that all alkyl-grafted RGO membranes can efficiently separate a 10 vol% water/alcohol mixture by direct filtration, allowing alcohol but inhibiting water to pass through, to produce a permeate of more than 97 vol% alcohol (Table 1).

**Table 1:** Aqueous mixtures (vol%) after separation via alkyl chain-modified RGO membranes with different thicknesses.

**Sample**	**Thickness (μm)**	**Water content in permeate (vol%)**				
		**Methanol**	**Ethanol**	**n-Propanol**	**i-Propanol**	**DMF**
C_3_H-RGO	0.69	3.11 ± 0.14	2.01 ± 0.07	1.51 ± 0.04	2.85 ± 0.11	2.37 ± 0.04
	0.89	1.82 ± 0.06	1.54 ± 0.05	1.39 ± 0.02	2.43 ± 0.06	2.72 ± 0.03
	0.93	1.42 ± 0.04	1.33 ± 0.03	1.20 ± 0.03	2.40 ± 0.04	2.53 ± 0.06
C_8_H-RGO	0.21	2.51 ± 0.07	1.73 ± 0.05	1.37 ± 0.04	2.03 ± 0.04	2.44 ± 0.14
	0.42	1.41 ± 0.04	1.16 ± 0.04	0.94 ± 0.02	2.02 ± 0.03	2.27 ± 0.08
	0.58	1.12 ± 0.02	1.12 ± 0.02	0.09 ± 0.01	1.89 ± 0.03	2.15 ± 0.06
C_12_H-RGO	0.18	1.85 ± 0.06	1.67 ± 0.04	0.74 ± 0.01	1.98 ± 0.08	2.03 ± 0.07
	0.24	1.22 ± 0.03	1.04 ± 0.02	0.68 ± 0.01	1.71 ± 0.06	1.89 ± 0.05
	0.49	0.90 ± 0.01	0.68 ± 0.01	0.04 ± 0.01	1.10 ± 0.03	1.72 ± 0.05
RGO^a^	0.36	10	10	10	10	10

a *Obtained by hydrazine reduction*.

For all alkyl-RGO membranes with different thicknesses, after the separation of methanol/water, ethanol/water and n-propanol/water mixtures, the water contents in the permeate are in the order of methanol > ethanol > n-propanol. The increased separation efficiency is attributed to the alkyl chain interaction between the alcohols and the modified RGO membranes, where longer alkyl chains of the alcohol (e.g., n-propanol) lead to higher interaction with the alkyl-RGO membranes. For example, by using C_3_H-RGO membrane with a thickness of 0.93 μm, the water content in the permeate decreases from 1.42 ± 0.04 vol% for methanol/water mixture to 1.20 ± 0.03 vol% for n-propanol/water mixture. In addition, for all the water/alcohol mixtures, the separation efficiency also increases as the alkyl-RGO membrane changes from C_3_H-RGO to C_12_H-RGO, which is also interpreted as due to increased alkyl chain interactions. For example, the water content can be decreased to 0.04 ± 0.01 vol% after separation via a C_12_H-RGO membrane with a thickness of 0.49 μm. The separation factor of C_12_H-RGO membrane (0.49 μm) for separating n-propanol/water is about 278, which is higher than that of the reported membrane for separation of water/alcohol via the pervaporation process (Tang et al., 2014; Igi et al., 2015). However, for the mixtures with higher water concentrations, the separation efficiency reduces. For example, after separation of the n-propanol/water mixtures with 20% volume fraction of water, the water content in the permeate is about 1.5 vol% and the separator factor is calculated to be about 16.

In addition, to confirm the effectiveness of water/alcohol separation, we measured the water CA and alcohol CA of pure cellulose ester membrane and GO deposited cellulose ester membrane. For the two kinds of membranes, they all behave hydrophilic and oleophilic. When water droplet was dropped on the GO membrane, it penetrated into the membrane within 40 s (Supplementary Figure 5). When water/alcohol mixtures were poured on the GO membranes, the water/alcohol mixtures were permeated the membrane together and could not be separated (Supplementary Figure 6). Geim et al. (Nair et al., 2012) reported that thick RGO membranes (≈0.5–1.0 μm) were impermeable to all molecules including water. Remarkably, in our case, modification and reduction of GO offer a much more straightforward approach to control the passages for alcohol and water. To further confirm the role of alkyl chains on the RGO sheets, we prepared a RGO membrane by hydrazine hydrate. The water contact angle of the RGO membrane decreases with time, and the oil droplet (e.g., hexane) immediately penetrates into the membrane after touching the RGO surface, indicating the hydrophilic and oleophilic properties of the RGO membrane (Supplementary Figure 7). After filtration of water/alcohol miscible solution, the film reveals no separation ability for all miscible solutions as confirmed by GC.

It should be mentioned that not only the alkyl chain length of the alcohol but also the structure of the alcohol play a role in affecting the separation efficiency. As shown in Table 1, the water content after separation of i-propanol/water for all of the alkyl-RGO membranes is slightly higher than that of the permeate after separation of the n-propanol/water mixture. In addition, the separation efficiency of DMF/water miscible solution was also checked (Table 1). The water content in the permeate is decreased as the alkyl chain length increases and reaches 1.72 ± 0.05 vol% after separation by the C_12_H-RGO membrane.

It is noted that the whole separation process is only driven by gravity without any other external force. All miscible solutions could be successfully separated in one step. Although the diameter of alkyl-RGO membranes is 4 cm in this study due to the limitation of filtration setup, they could be larger according to the actual situation. The fluxes of the permeate through the RGO-based membrane were determined by measuring the time for almost completely permeating a certain volume of the solution. For C_8_H-RGO and C_12_H-RGO membranes, the fluxes decrease with the increase in the thickness of the membranes (Table 2). Permeation theory predicts that the filtration rate is directly proportional to the square of the effective pore size of the membrane and inversely proportional to the thickness of the membrane (Peng et al., 2009). In our case, the observed data are consistent with the separation theory in that a thicker membrane will sacrifice its effective pore size, resulting in a slower filtration rate. However, the fluxes do not always decrease as the thickness of the alkyl-RGO membrane increases. For example, for the C_12_H-RGO membrane during the separation of all water miscible solutions, the fluxes of the permeates decrease first and then show almost no change as the thickness of the membrane increases (Supplementary Figure 8).

**Table 2:** Permeate flux of different water miscible liquids.

**Sample**	**Thickness (μm)**	**Permeate flux (mL m**^****−2****^ **h**^****−1****^**)**				
		**Methanol**	**Ethanol**	**n-Propanol**	**i-Propanol**	**DMF**
C_3_H-RGO	0.69	80 ± 9	63 ± 7	19 ± 5	15 ± 4	346 ± 35
	0.89	316 ± 23	342 ± 32	350 ± 18	125 ± 15	780 ± 41
	0.93	261 ± 21	336 ± 27	318 ± 16	120 ± 12	855 ± 38
C_8_H-RGO	0.21	396 ± 35	445 ± 43	310 ± 33	246 ± 17	664 ± 41
	0.42	286 ± 21	386 ± 31	187 ± 22	119 ± 15	585 ± 31
	0.58	350 ± 31	367 ± 25	167 ± 18	105 ± 16	453 ± 36
C_12_H-RGO	0.18	1386 ± 54	1533 ± 50	943 ± 26	1027 ± 51	1289 ± 51
	0.24	1226 ± 45	1335 ± 37	740 ± 22	852 ± 36	1134 ± 42
	0.49	1035 ± 38	1138 ± 31	685 ± 31	734 ± 31	923 ± 34

It should be noted that not only the thickness of the membrane but also the viscosity of the organic liquid affect the fluxes of the permeate. Usually, the flux is in inverse proportion to liquid viscosity (Han et al., 2013). As shown in Table 2, for C_12_H-RGO membrane with a fixed thickness of 0.49 μm, the fluxes of methanol and ethanol are 1,035 ± 38 and 1,138 ± 31 mL m^−2^ h^−1^, which are much higher than that of propanol and isopropanol. This may be due to the higher viscosity of propanol (2.26 mPa s) and isopropanol (2.43 mPa s) than that of methanol (0.55 mPa s) and ethanol (1.07 mPa s). For DMF with a viscosity of 0.92 mPa s, a flux of 923 mL m^−2^ h^−1^ is obtained.

After reduction, the interplanar distance of all alkyl-RGO is larger than the kinetic diameter of water (0.265 nm), namely, water can pass through the spacing (Huang et al., 2014). However, water is blocked for all alkyl-RGO membranes, which suggests that molecular sieving is not the major mechanism for water/alcohol separation. The successful separation of water miscible solution by the filtration process relies on the superhydrophobic interaction between the alkyl chain on the RGO sheet and alcohols and the selective absorption, as well as the diffusion rate difference of the alcohols through the membrane.

### Molecular Dynamic Simulations

**Figure 6:**
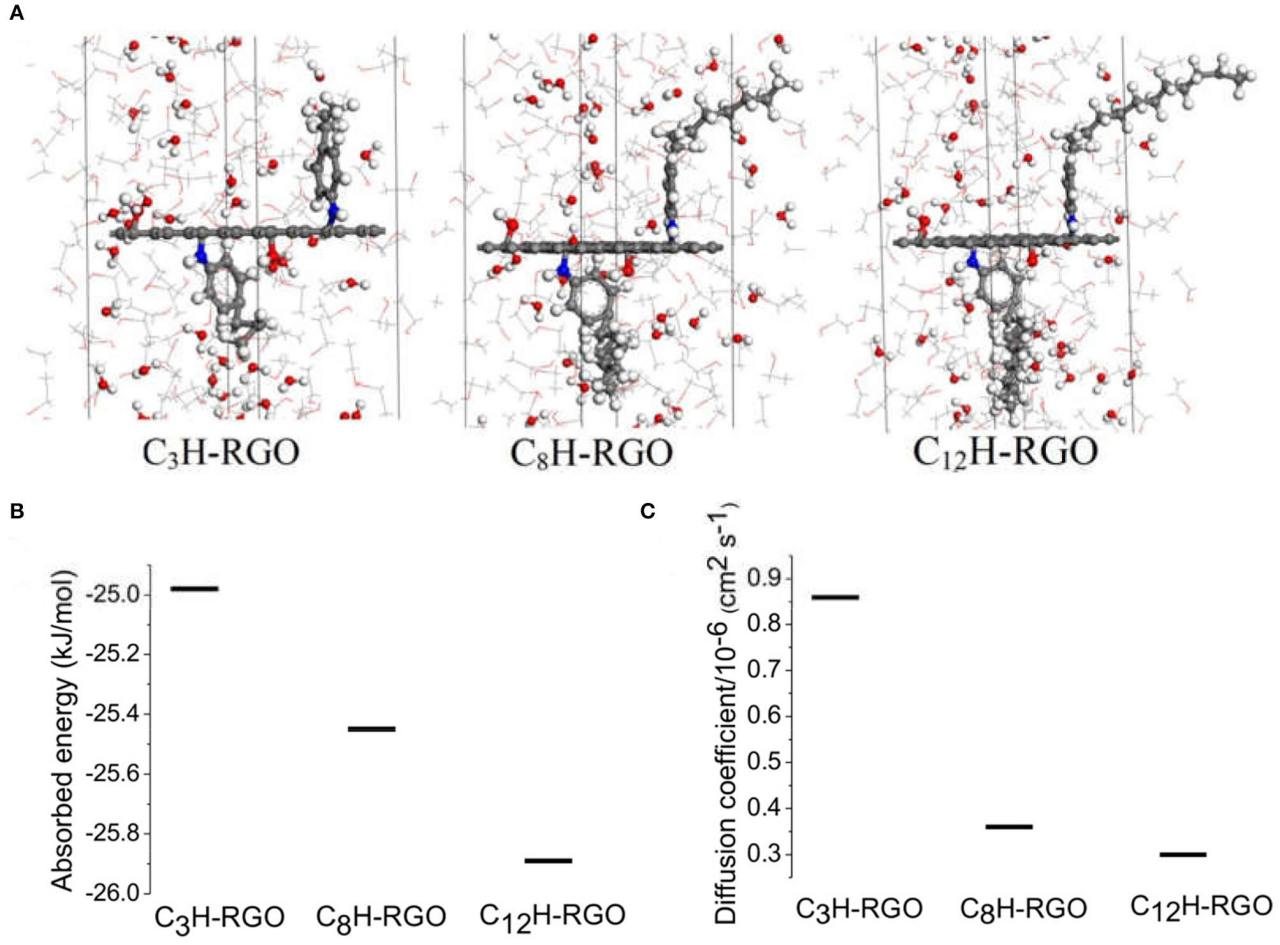
Molecular dynamic simulation. **(A)** A single layer of alkyl-RGO is placed in the middle of the ethanol aqueous solution, Line: ethanol molecules; ball: water molecules; for the elements: red: O; gray: C; white: H; blue: N. **(B)** Absorbed energy of ethanol molecule and **(C)** diffusion coefficient of water molecules on the alkyl-RGO sheet.

To further prove the absorption and diffusion difference in the alcohol and water through the membrane, a series of molecular dynamics simulations were performed. Here, we selected ethanol as a model. As shown in Figure 6A, three different single layer systems (C_3_H-RGO, C_8_H-RGO, and C_12_H-RGO) with the same concentrations of ethanol aqueous solution were placed in the middle of the ethanol aqueous solution. The ethanol aqueous solution for each system consists of 200 ethanol molecules and 70 water molecules in which the concentration is the closest to our experiments. All of the systems were simulated in the cubic simulation lattice built with dimensions of x = 9.8 Å, y = 12.3 Å, and z = 102 Å. Detailed simulation methods are described in the Experimental section. From an energy aspect, the absorbed energy of ethanol on a single layer of alkyl-RGO is calculated to be −24.98 kJ/mol for C_3_H-RGO, −25.45 kJ/mol for C_8_H-RGO, and −25.89 kJ/mol for C_12_H-RGO (Figure 6B, Supplementary Table 2). This means that ethanol molecules can be easily absorbed on the alkyl-RGO surface in the order of C_12_H-RGO > C_8_H-RGO > C_3_H-RGO. Many effective analytical methods can estimate the diffusion behavior of small molecules, like alcohol (Yang and Lue, 2013), in membrane materials. Considering the characteristics of our simulation system, we finally calculated the diffusion coefficients to analysis the diffusion behavior of water and ethanol molecules. The diffusion coefficient of water on the surface of alkyl-RGO is also analyzed to quantify the affinity between the alkyl-RGO and water molecule, which is calculated to be 0.86 × 10^−6^ cm^2^ s^−1^ for C_3_H-RGO, 0.36 × 10^−6^ cm^2^ s^−1^ for C_8_H-RGO, and 0.30 × 10^−6^ cm^2^ s^−1^ for C_12_H-RGO (Figure 6C, Supplementary Table 3). A much lower diffusion coefficient of water molecules indicates that alcohol passes through the C_12_H-RGO membranes more easily. In addition, to further prove the successful separation of alcohol and water, thermodynamic simulation was also performed as shown in Supplementary Material. After separation, the sum energy of the alcohol and water is obviously lower than that of the water/alcohol mixtures.

## Conclusion

Different alkyl chain-grafted RGOs were obtained by simultaneous grafting and reduction of GO. The alkyl-RGO membranes obtained by vacuum filtration can be used to separate water/oil immiscible mixtures. More importantly, the membranes can be used to separate water/alcohol miscible solutions. The alkyl chains on the RGO rendered the alkyl-RGO membrane more hydrophobic and facilitated the alcohol passing through the membrane while blocking water penetration. Molecular simulation indicated that the selective absorption ability and diffusion rate affected the water/alcohol separation. Although the mechanism of filtration needs to be deeply investigated, the separation of water/alcohol miscible mixtures driven solely by gravity is undoubtedly an alternative compared to the pervaporation technology for water/alcohol separation.

## Data Availability Statement

The original contributions presented in the study are included in the article/Supplementary Material, further inquiries can be directed to the corresponding authors.

## Author Contributions

LL and ZX designed and wrote the whole work. HZ and JY did the most of the work. XJ characterized the work. TL and YZ analyzed the work. All authors named on the manuscript have made a significant contribution to the writing, concept, design, execution, or interpretation of the work represented. All authors agree with the authors list appeared on the manuscript.

## Conflict of Interest

The authors declare that the research was conducted in the absence of any commercial or financial relationships that could be construed as a potential conflict of interest.
