# Supplementary material for: Alkyl Chain Grafted-Reduced Graphene Oxide Membrane for Effective Separation of Water/Alcohol Miscible Mixtures
Source: Front Chem. 2020 Dec 3;8:598562. doi: 10.3389/fchem.2020.598562 (PMC7744741; doi:10.3389/fchem.2020.598562)
Supplement: Supplementary file 1 [file Table_1.DOCX]

Supporting information

Alkyl-Chain Grafted-Reduced Graphene Oxide Membrane for Effective Separation of Water/Alcohol Miscible Mixtures

Hailong Zhang^#^, Jianbo Yang^#^, Ting Li, Xingxiang Ji, Zhen Xu*, Yaling Zhu, Libin Liu*

State Key Laboratory of Biobased Material and Green Papermaking, School of Chemistry and Chemical Engineering, Qilu University of Technology (Shandong Academy of Sciences), Jinan 250353, China

^#^ These authors contributed equally: H. Zhang, J. Yang.

*Corresponding author. E-mail: [lbliu@qlu.edu.cn](mailto:lbliu@qlu.edu.cn) (Libin Liu), [xuzhen123@126.com](mailto:xuzhen123@126.com) (Zhen Xu)

Preparation of graphene oxide (GO)

Graphene oxide was prepared from natural graphite by the Hummers method. In brief, 36 ml of sulfuric acid was added to a 250-ml round-bottom flask placed in an ice-bath. About 1 g of natural graphite and 0.75 g of NaNO_3_ were dispersed in the cold sulfuric acid with a magnetic stirrer. Subsequently, 5 g of KMnO_4_ was added slowly over 15-20 min. After that, the reaction mixture was kept at 40 ℃ for 30 min and the resulting mixture was stirred for 2 h. The temperature of the water bath was increased to 80 ℃ and the reaction was continued for another 1 h in order to increase the degree of oxidation of the graphite. Then, the temperature was decreased to room temperature. The resultant suspension was diluted with 50 ml of deionized water and further treated with H_2_O_2_ solution until the colour changed to bright yellow. The bright yellow product was washed several times with 10% HCl. The unexfoliated graphite oxide was removed by centrifugation. Finally, graphene oxide (GO) was obtained through sonication for 1 h.


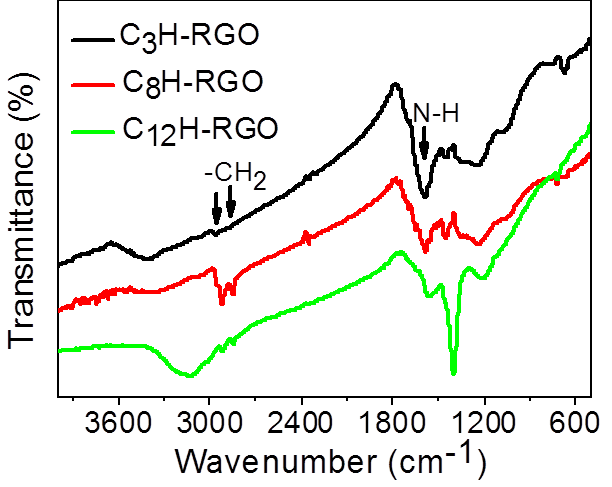


Figure S1. FTIR spectra of alkyl chain-modified RGO.


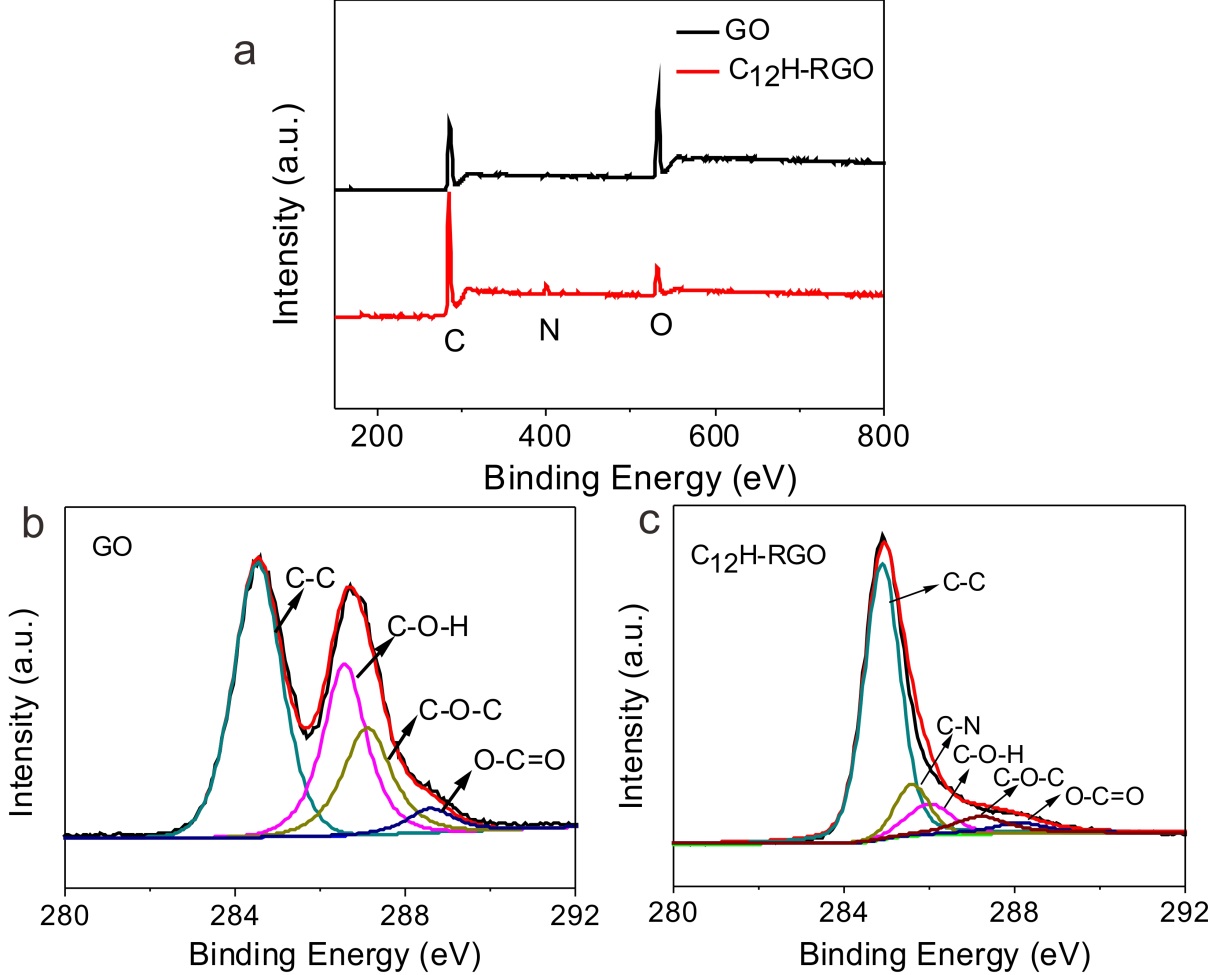


Figure S2. XPS spectra of GO and C_12_H-RGO.


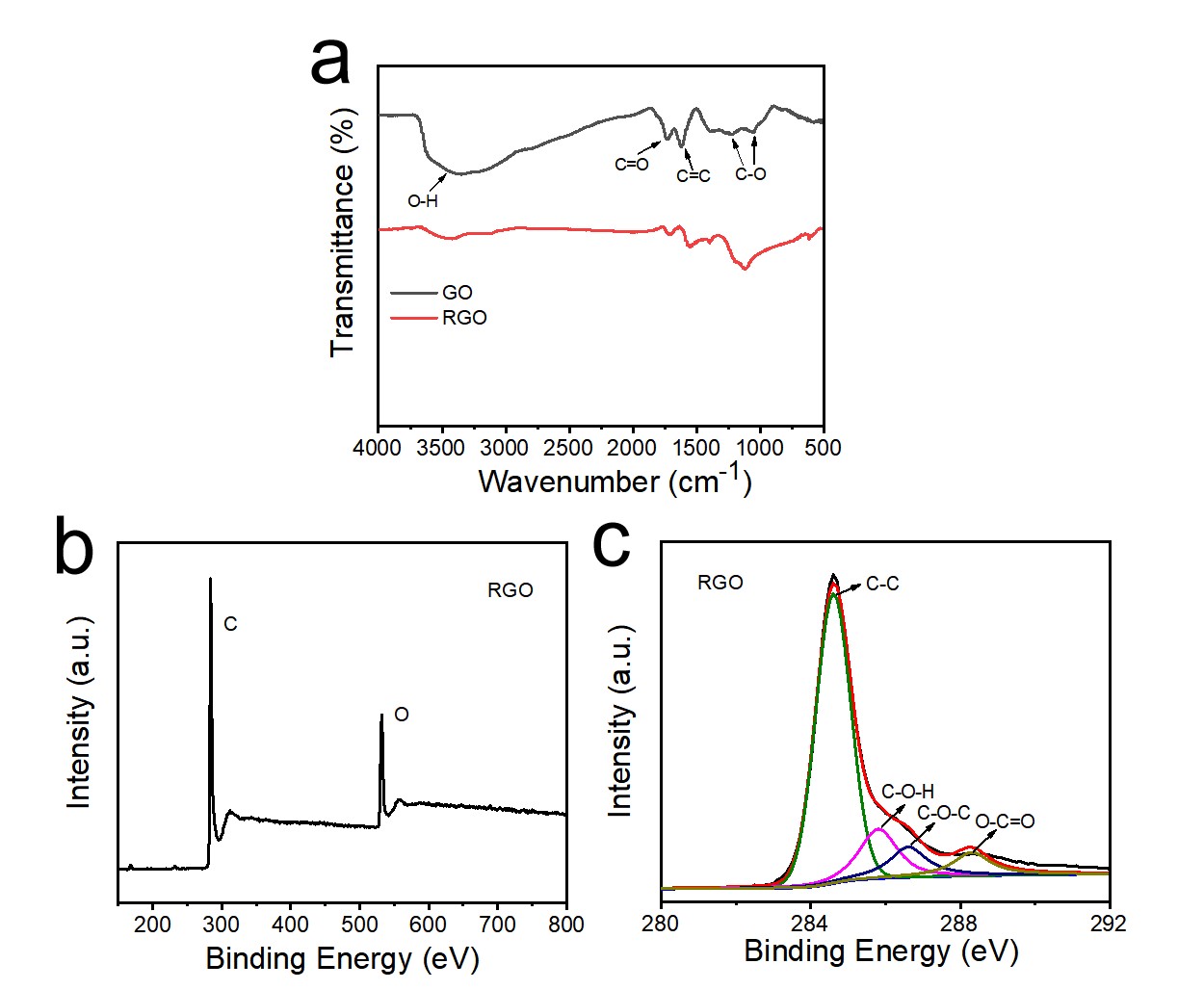


Figure S3. FTIR spectra (a) and XPS spectra (b, c) of RGO obtained by hydrazine reduction.

When GO was reduced by hydrazine, the oxygen-containing groups on GO sheets are highly decreased as shown in FTIR spectra (Figure S3a). The XPS spectra reveal that the molar ratio of C/O is 5.2 for RGO which is slightly high than that of C12-RGO (4.2). The C 1s spectra of RGO also reflected the reduction process. For GO, four different peaks centered at 284.6, 286.6, 287.2, and 288.5 eV correspond to C–C in the unoxidized graphite carbon skeleton, C–OH in the hydroxyl group, C–O–C in the epoxide group, and O–C=O in the carboxyl group, respectively. After reduction by hydrazine, the peaks corresponding to the oxygen-containing groups of RGO are significantly weakened, indicating that a large number of oxygen-containing groups have been removed (Figure S3b,c).


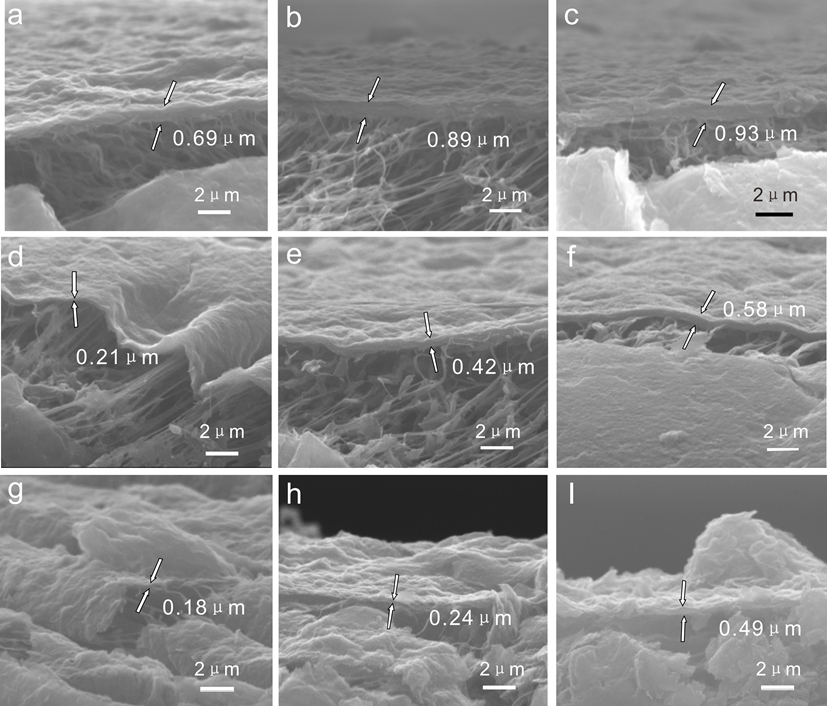


Figure S4. SEM images of different thicknesses of alkyl chain-modified RGO: (a, b, c) C_3_H-RGO membranes, (d, e, f) C_8_H-RGO membranes, (g, h, i) C_12_H-RGO membranes.

Table S1. Properties of the organic liquids used.

| Liquids | Density (g cm^-3^) |  |
| --- | --- | --- |
| Diesel | 0.850 |  |
| Petroleum ether | 0.75 |  |
| Toluene | 0.863 |  |
| n-Hexane | 0.656 |  |
| Bromoethane | 1.47 |  |
| Dichloromethane | 1.33 |  |


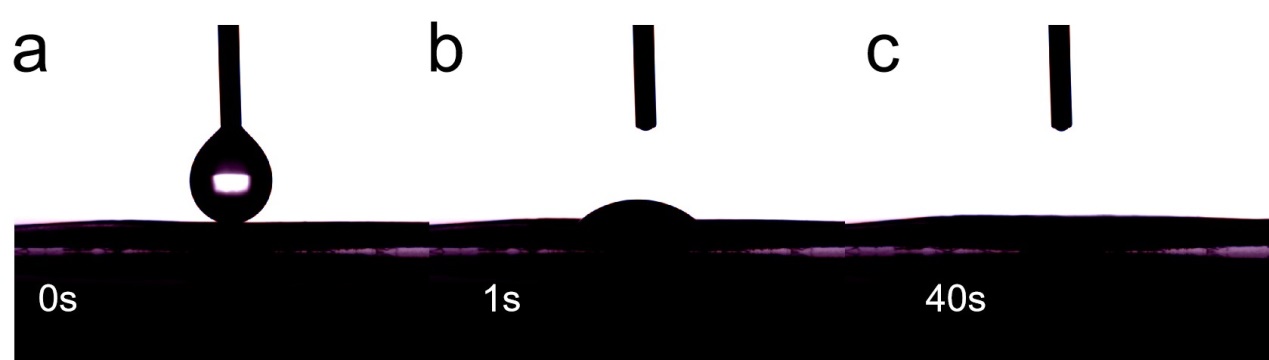


Figure S5 Water droplet penetrated into the GO membrane within 40s.


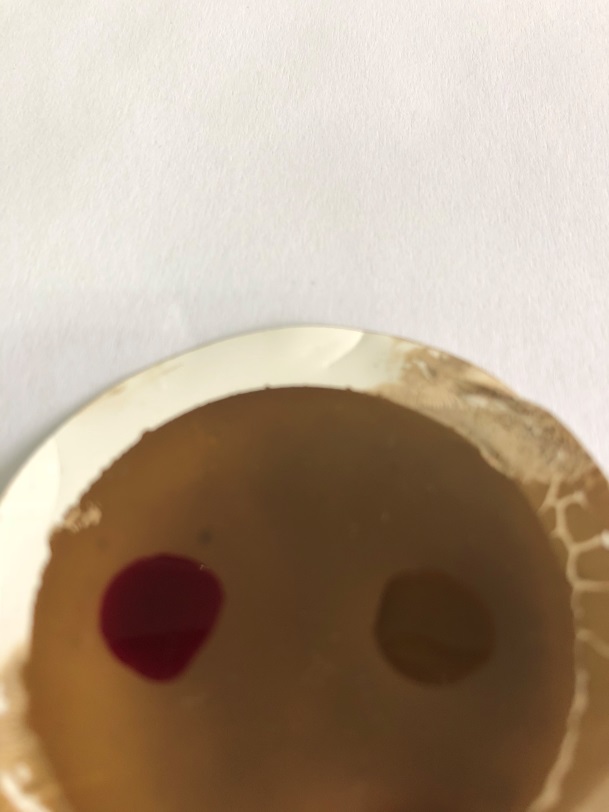


Water

Water/alcohol

Figure S6. Photograph of water and water/alcohol (colored in red) mixtures absorbing into GO membrane within 1 second.


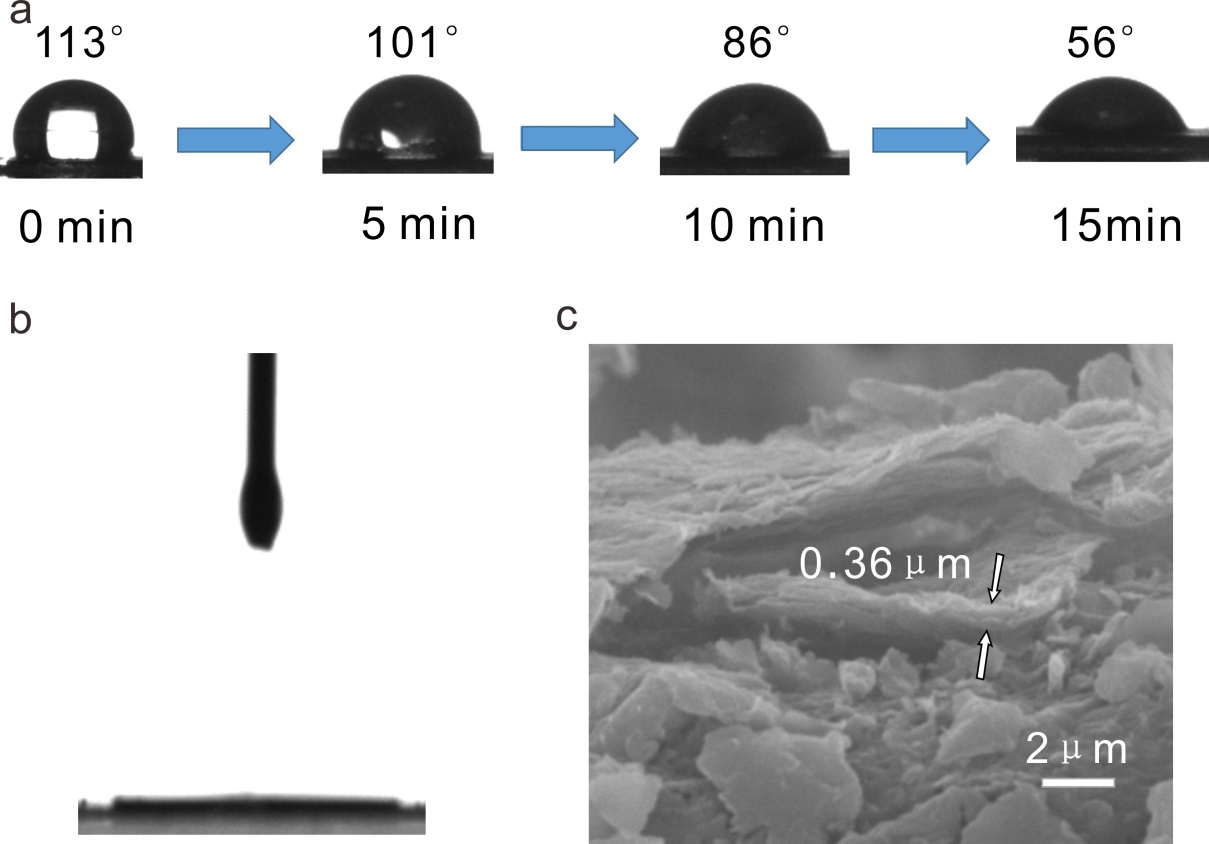


Figure S7. Water contact angle (a) and oil contact angle (b) of RGO membrane; (c) a RGO membrane with a thickness of 0.36 μm for testing oil/water separation.


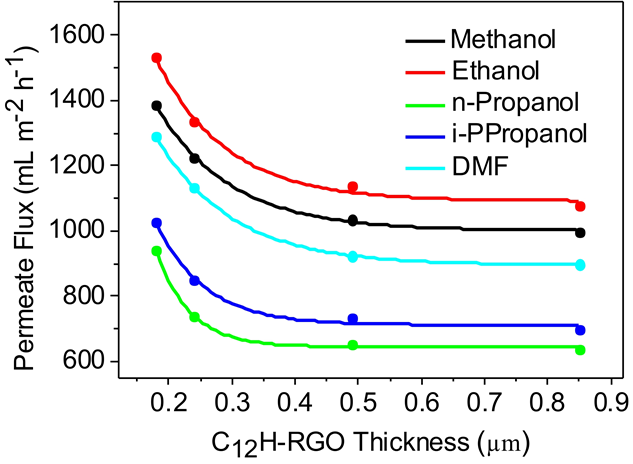


Figure S8. Permeate flux of C_12_H-RGO with different membrane thicknesses.

**Table S2.** Absorbed energy of the water and alcohol molecules during the simulation

| System | C_3_H-RGO | C_8_H-RGO | C_12_H-RGO |
| --- | --- | --- | --- |
| E water（kJ/mol） | -24.58 | -24.33 | -23.39 |
| E alcohol（kJ/mol） | -24.98 | -25.45 | -25.89 |

**Table S3.** Self-diffusion coefficients of the water and alcohol molecules during the simulation

| system | D(water) /10^-6^ cm^2^ s^-1^ | D (alcohol) /10^-6^ cm^2^ s^-1^ |
| --- | --- | --- |
| C_3_H-RGO | 0.86 | 0.78 |
| C_8_H-RGO | 0.36 | 0.88 |
| C_12_H-RGO | 0.30 | 0.89 |

**Characterization of the water content after separation of water/alcohol miscible solution by the gas chromatography (GC) technique**

Ethanol/water miscible solution is used as an example.

(1) Draw the standard curve.

Different volumes of ethanol are dissolved in DMF, and cyclohexanone is used as the internal standard substance. The integral area ratio of S_Ethanol_/S_Cyclohexanone_ (S_E_/S_C_) is measured by controlling the volume ratio of V_Ethanol_/V_Cyclohexanone_ (V_E_/V_C_). For example, different volumes of ethanol and cyclohexane are dissolved in DMF (10 mL). The integral area of ethanol (S_E_) and cyclohexanone (S_C_) could be obtained by measuring GC as shown in Table S4. Then, the standard curve could be drawn (Figure S9).

(2) Measure the water content in the permeates.

After separation of the ethanol/water miscible solution, the permeate (e.g., 0.1 mL) is added in DMF (10 mL) with cyclohexane (0.1 mL) as the internal standard substance. The integral area of ethanol and cyclohexanone is measured by GC (Figure S10). According to the standard curve, the volume ratio of ethanol and cyclohexanone can be obtained as 0.98. Thus, the volume of ethanol can be obtained (V_E_=0.098 mL). Finally, the water content in the permeate can be calculated as V_water_/V_permeate_ = 1-V_E_/V_permeate_ = 1-0.098/0.1 = 2 vol%.

Table S4. Integral area of ethanol (S_E_) and cyclohexanone (S_C_)

| DMF (V/mL) | Ethanol (V/mL) | S_E_ | Cyclohexanone (V/mL) | S_c_ | V_E_/V_C_ | S_E_/S_C_ |
| --- | --- | --- | --- | --- | --- | --- |
| 10 | 0.05 | 338954 | 0.1 | 1211423 | 0.5 | 0.28 |
| 10 | 0.1 | 558387 | 0.3 | 2440500 | 0.3 | 0.22 |
| 10 | 0.2 | 913339 | 0.2 | 1682971 | 1 | 0.54 |
| 10 | 0.3 | 1385329 | 0.1 | 919512 | 3 | 1.51 |


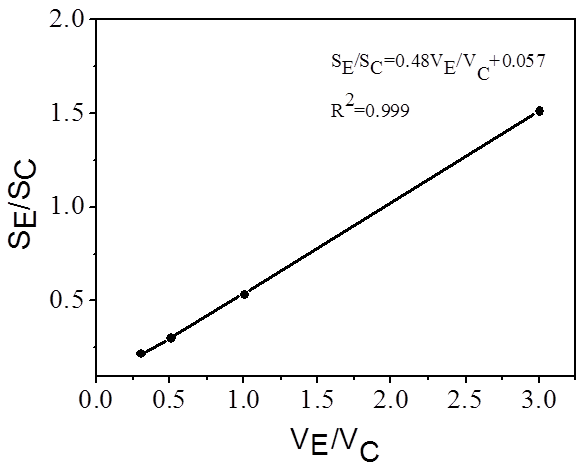


Figure S9. The standard curve by using pure ethanol.


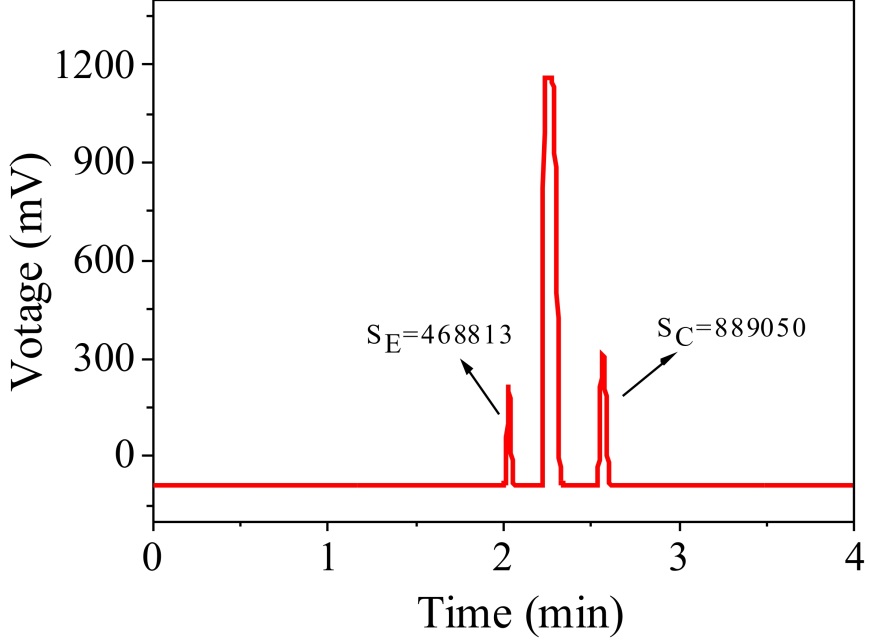


Figure S10. Integral area of ethanol (SE) and cyclohexanone (SC) in the permeate after separation of the ethanol/water miscible solution.

**Thermodynamic simulation**

To provide credible evidence from a thermodynamic view, we have designed three theoretical models similar to our experiments. Firstly, 10 water molecules were randomly dissolved in 30 ethanol molecules to form system 1 in a cubic lattice using the Amorphous Cell module, Materials Studio 2018 (Accelrys Inc) software. Subsequently, all molecules in system 1 are specifically divided into two systems: system 2 was composed of 8 water molecules and 10 ethanol molecules, and system 3 was composed of 2 water molecules and 20 ethanol molecules. The initial cubic simulation lattice was the same for all three systems, build with the dimensions (x = 14.8 Å, y= 14.8 Å, and z= 14.8 Å). The simple point charge (SPC) model ^[1,2]^, which can accurately describe the water solution environment ^[3]^, is adopted for all water molecules.

The energies of the 3 initial conﬁgurations were minimized with the Smart Minimizer method. After the minimization, all molecular dynamic simulations were equilibrated at constant temperature (298.15 K) and volume (NVT) for 10 ns. Atomic coordinates were saved for every 200 ps. The snapshot of the last 200 ps was adopted as table structures for the 3 systems. The MD simulation was performed after charges and potentials were assigned to each atom. The long-range electrostatic interactions have been accounted for using the Ewald method. The total energy is written as a combination of valence terms including diagonal and off-diagonal cross-coupling terms and nonbond interaction terms, which include the Coulombic and Lennard-Jones functions for electrostatic and van der Waals interactions,

$E=E_{bonds}+E_{angles}+E_{dihedrals}+E_{cross}+E_{VDW}+E_{elec}$ (1)

where E_VDW_ and E_elec_ are given by the eq 2:

$E_{non-bond}=E_{VDW}+E_{elec}=\sum\varepsilon_{ij}\left[2\left(\frac{\sigma_{ij}}{r_{ij}}\right)^{9}-3\left(\frac{\sigma_{ij}}{r_{ij}}\right)^{6}\right]+\sum\frac{q_{i}q_{j}}{r_{ij}}$ (2)

The parameters for each like-site interaction are given by the COMPASS force field ^[4,5]^.

As shown in Figure S11, the sum energy of system 2 and 3 (-146.079 Kcal/mol) is obviously lower than that of system 1 (-134.944 Kcal/mol). This result fully proves that this process of separating system 1 into systems 2 and 3 is a process approaching more stable from the view of thermodynamic energy. The energies listed in Figure S11 are the energies of the final stable configurations in our MD process, which are calculated from formulas 1 and 2. These energy data can also be calculated from thermodynamic formula (*U = H - pV*) in the classical physical chemistry textbook. The results are the same. All the original thermodynamic data for our MD simulations are also listed in Table S5-7.


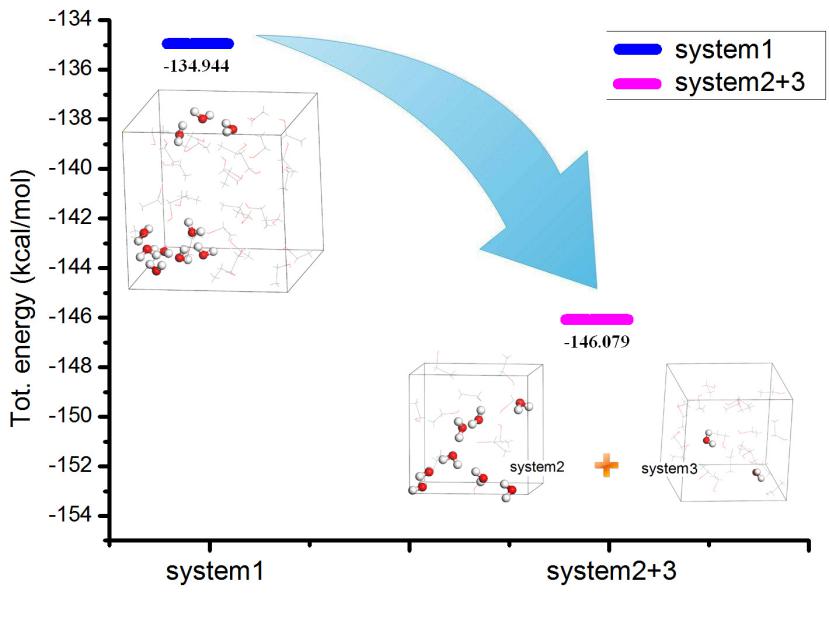


Figure S11. The evolution process of energy change for the system1 and system 2+3 (unit Kcal/mol)

**Table S5. The original thermodynamic data of MD simulations for system 1.**


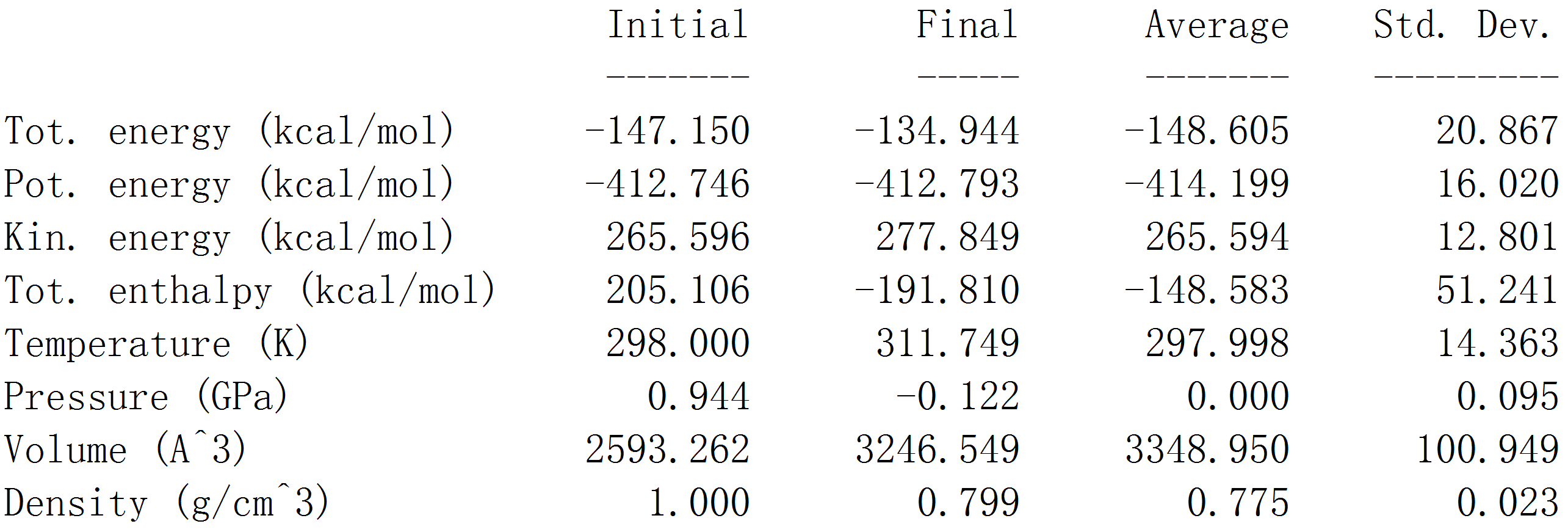


**Table S6. The original thermodynamic data of MD simulations for system2.**


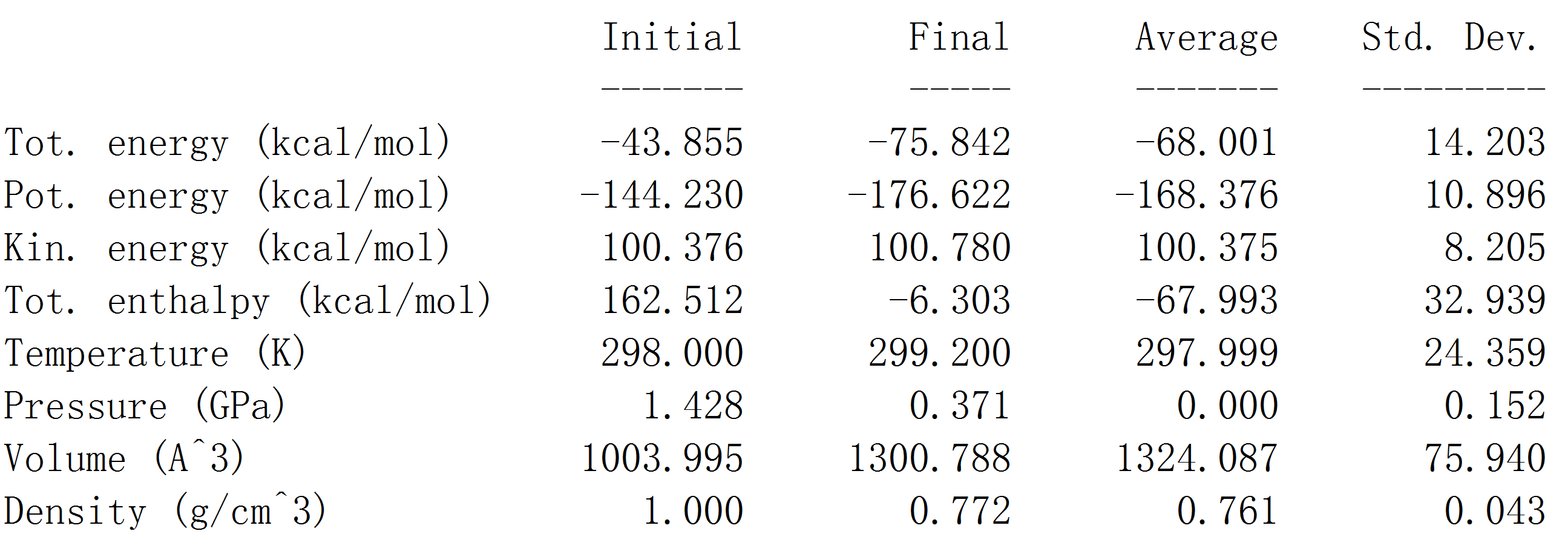


**Table S7. The original thermodynamic data of MD simulations for system3.**


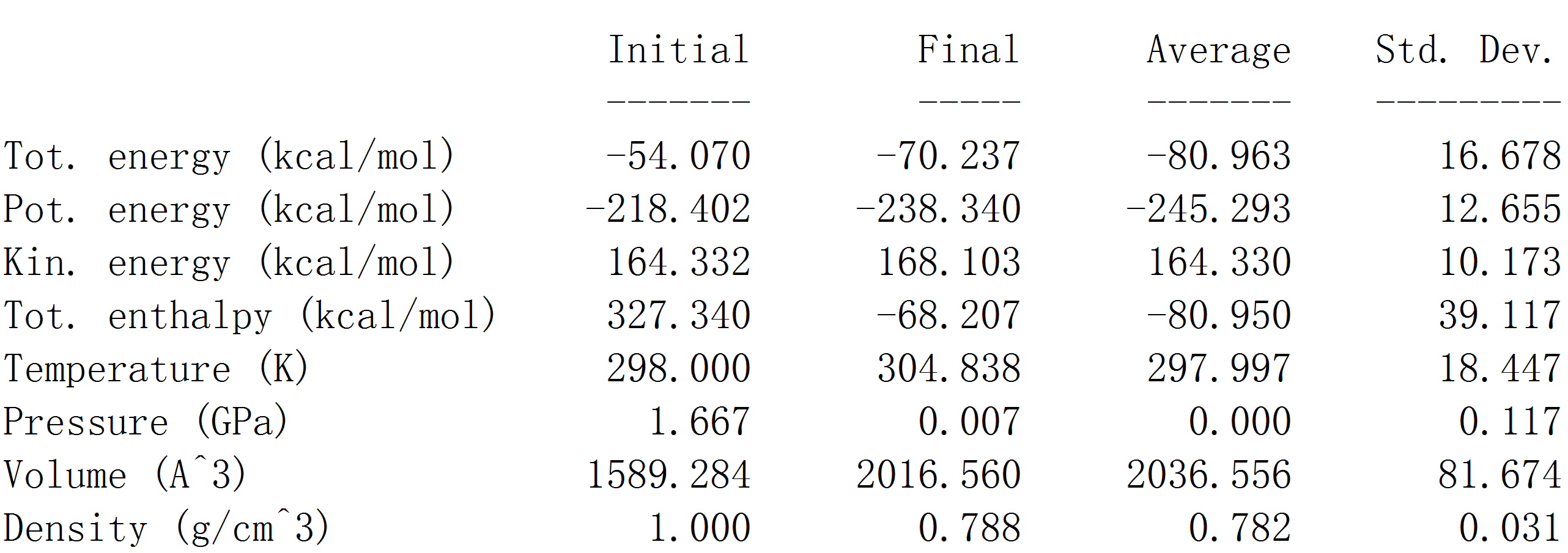


**References**

[1] H. J. C. Berendsen, J. P. M. Postma, W. F. van Gunsterenand J. Hermans, in Intermolecular Forces, ed. B. Pullman,Reidel Publ., Dordrecht, 1981, pp. 331–342.

1. H. J. C. Berendsen, J. R. Grigera and T. P. Straatsma, The missing term in effective pair potentials, J. Phys. Chem., 1987, 91(24), 6269–6271.
2. J. Wiedemair Martin, S. Hofer Thomas, Towards a dissociative SPC-like water model-probing the impact of intramolecular Coulombic contributions, Physical Chemistry Chemical Physics., 2017, 19, 31910-31920.

[4] H. Sun, P. Ren, J. R. A. libai and JG simmons, The compassforce field: parameterization and validation for phosphazenes, Comput. Theor. Polym. Sci., 1998, 8, 229-246.

[5] H. Sun, [COMPASS: an ab initio force-field optimized for condensed-phase applicationsoverview with details on alkane and benzene compounds](http://pubs.acs.org/doi/abs/10.1021/jp980939v?prevSearch=), J. Phys. Chem. B, 1998, 102, 7338-7364.
